# Management of traumatic hemipelvectomy: an institutional experience on four consecutive cases

**DOI:** 10.1186/1757-7241-21-64

**Published:** 2013-08-16

**Authors:** Tian-hao Wu, Xi-rui Wu, Xiao Zhang, Chun-sheng Wu, Ying-ze Zhang, A-qin Peng

**Affiliations:** 1 Emergercy Trauma Center, Third Hospital of Hebei Medical University, Shijiazhuang 050051, China

**Keywords:** Trauma, Hemipelvectomy, Damage control

## Abstract

**Background and objective:**

The incidence of traumatic hemipelvectomy is rare, but it is a devastating injury. Recently, an increasing number of patients with traumatic hemipelvectomy are admitted to trauma centers alive due to improvements of the pre-hospital care. Successful management requires prompt recognition of the nature of this injury and meticulous surgical technique. We present our successful experiences on four cases of traumatic hemipelvectomy in the past nine years.

**Patients and methods:**

Four cases with traumatic hemipelvectomy were admited to our hospital from June 21, 2002 to September 3, 2011. All injuries occurred due to vehicle accident and all patients were in a state of severe hypotension, with two of them having anal lacerations. These four cases were treated immediately with resuscitation, control of hemorrhage, early amputation, repeated debridement and closure of the wounds. An angiographic embolization was given to control hemorrhage in two of the cases preoperatively. One case underwent fecal diversion. Wound infection occurred in all of cases which was successfully controlled by repeated debridements, effective anti-biotic regimen, split-thickness skin grafts.

**Results:**

All four cases were saved successfully with well-healed wounds during follow up from 1 to 7 years. They were able to walk by themself using crutches.

**Conclusion:**

Adhering to the surgery principles of damage control including appropriate resuscitation, hemorrhage control, coagulopathy correction and multiple debridements and closure of the wounds in reasonable period of time can save the life of cases suffering from severe pelvic ring injury.

## Introduction

Traumatic hemipelvectomy is a catastrophic injury. It is rarely seen in clinical practice because only a small number of cases can be survival and are transfered to the hospital. From 1915, when the first case of traumatic hemipelvectomy was successfully treated [1], there have been increasing numbers of cases were reported to be successfully treated [2-4]. A total of seven cases with traumatic hemipelvectomy were treated in our hospital from June 21, 2002 to September 3, 2011. Two cases died from hemorrhage before and after operation respectively, and one case was transferred to a local hospital two days after amputation and was lost to follow-up. The remaining four cases were successfully treated. Here we report the treatment experience on these four cases and summarize a literature review for the management of traumatic hemipelvectomy.

### Case 1

**Figure 1:**
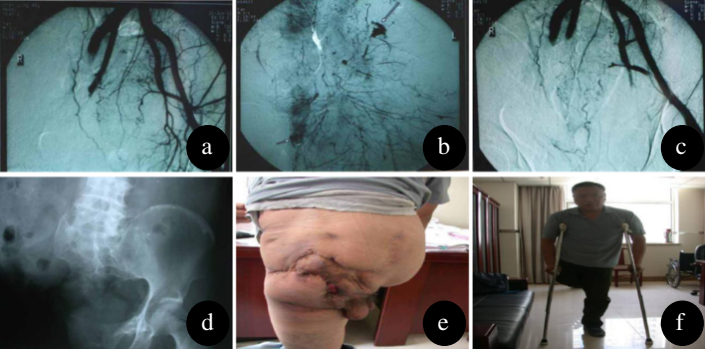
**Male, 45 years old, admitted for right hemipelvectomy, 12 h after he was involved in a traffic accident. (a)** The preoperative angiography showed that the right internal and external arteries were occluded, and the left ones were normal. Hemorrhage from the small arteries was observed. **(b)** The magnified picture in some portions shows active hemorrhage from the small arteries. **(c)** After angiographic embolization of the left internal iliac artery and right internal and external arteries, no hemorrhage was observed. **(d)**, **(e)**, **(f)** Radiography of the pelvis seven years after the procedure, the wound was healed, mild anal mucosal prolapse was noticed, the patient could ambulate with crutches.

A 45-year-old man, right lower limb being wedged in the rear wheel of a roller when walking across the street, was transferred to our hospital with his right lower limb unable to move for 12 h. Physical examination showing that the patient’s blood pressure was 85/57 mmHg and the pulse rate was 150 per minute. He was confusion and his right chest and rib area displayed extensive skin ecchymosis, with a wound area of 10 × 10 cm^2^ in the right hip that extended to the pelvis. There was a 3 cm laceration in his anal sphincter, and rigor mortis in the right lower extremity with cold skin and a loss of sensory and motor function. X-ray imaging showed that the right sacroiliac joint and pubic symphysis were separated, and the ilium was fractured. The patient was diagnosed with hemorrhagic shock, traumatic hemipelvectomy on the right side and laceration of the anal sphincter. Active resuscitation was performed. The blood pressure became stable and general condition was improved. But 8 hours later, the systolic pressure dropped persistently, and sustained hemorrhage in pelvis was suspected. The pelvic angiography was performed 12 hours after admission. It showed a wide range of small arterial bleeding in the pelvic cavity and a thrombosis in the right common iliac artery. The pelvic hemorrhage was significantly decreased following embolization of the left internal iliac artery. Due to the dead right lower extremity at the initial admission, hemipelvectomy was performed and the wound was covered with a gluteus maximus flap 33 h after admission. In view of the partial injury of anal sphincter, the mucosal eversion suture rather than a colostomy was performed. Following once debridement and twice skin grafting, the wound was healed. At the seven-year follow-up, the wound was in stable condition, his rectal mucosa was mildly prolapsed and the patient could walk with crutches (Figure 1).

### Case 2

**Figure 2:**
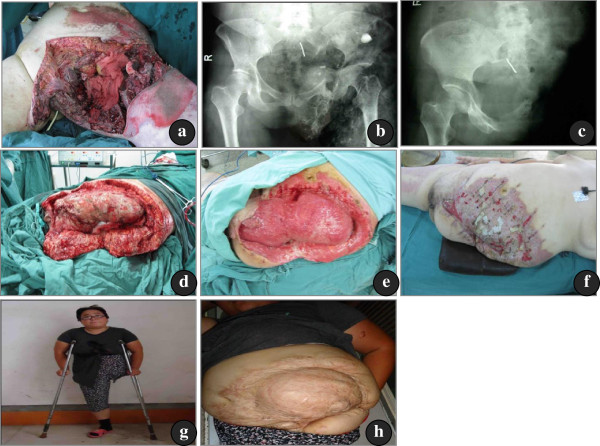
**(a), (b) Female, 35 years old, sustained traumatic hemipelvectomy in a traffic accident. (c)** The view after the amputation. **(d)** On the fourth postoperative day, severe soft tissue necrosis and wound infection was observed during the first debridement. On the 45th postoperative day, the first skin graft was performed. **(e)** &**(f)** On the 65th postoperative day, the second skin graft was performed. **(g)** &**(h)** the wound had healed and the patient could ambulate with crutches four years post-operation.

A 35-year-old female, who was hit by an oncoming car while cycling, was admitted to the hospital with her lower limb unable to move for 3 h. Physical examination indicated that her blood pressure was 84/45 mmHg and the pulse rate was 150 per minute. The patient was conscious, and had a wound area of 30 × 45 cm^2^ in the front of the left hip with extensive soft tissue injury. The pubic symphysis, parts of the ilium and femoral head were exposed. The lower left extremity was pale and pulseless with a complete loss of motor and sensory function. X-ray imaging showed left sacroiliac joint dislocation associated with pubic symphysis separation and dislocation of the left hip. Hemorrhagic shock and traumatic hemipelvectomy of the left side were diagnosed. Pressure dressing of the wound was applied during resuscitation. Hemipelvectomy was performed 3 hours after admission. The wounds were left open after the surgery because of the extensive soft tissue damage and contamination. Due to severe wound infection, debridement was required every 3 or 4 days following the hemipelvectomy. A total of six debridements and two skin grafting procedures were performed. She could walk with crutches 2 months after the injury. The wound healed completely 4 months after the injury. The phantom limb pain lasted for half a year and then resolved spontaneously. She has been followed up for 4 years after injury (Figure 2).

### Case 3

**Figure 3:**
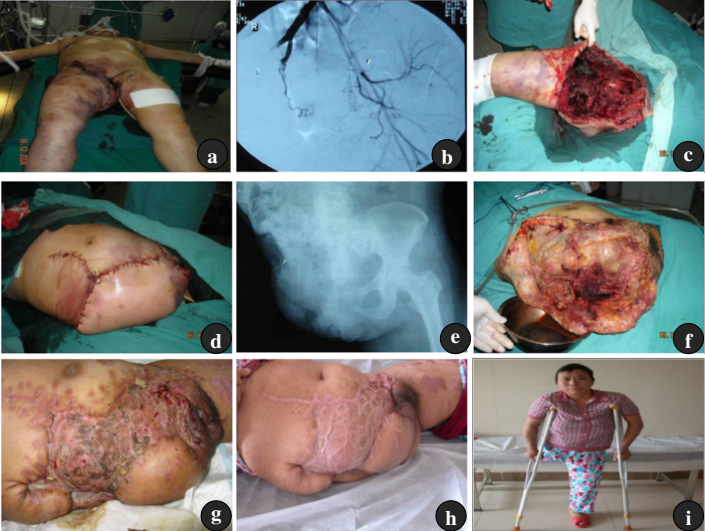
**Female, 40 years old, admitted to our hospital 12 h after the right lower extremity was injured by a truck. (a)** Resuscitation and the ligation of the external iliac artery were performed in the local hospital. **(b)** The preoperative angiography showed the external and partial internal iliac artery in the right were occluded, and no bleeding of the artery was observed. **(c)** The hemipelvectomy was performed 14 h after admission. **(d)** The wound was closed primarily. **(e)** Postoperative X-ray imaging. **(f)** On the third postoperative day, partial necrosis of the skin and severe wound infection was observed during the first debridement. **(g)** By the second postoperative month, the majority of wound had healed. **(h)** &**(i)** By the 12th postoperative month, the wound had healed, and the patient could ambulate with crutches.

A 41-year-old female, who was crushed from behind by a car while cycling, was admitted to our hospital with a bleeding wound in the medial of the right hip and the right lower limb unable to move for 12 h. Because her blood pressure was undetectable on admission to the local hospital, she received a transfusion of 6 units of red blood cells (RBCs). After ligation of the right external iliac artery and vein and wound suturing in the emergency room, the patient was transferred to our hospital. Physical examination indicated that the patient’s blood pressure was 138/70 mmHg and pulse rate was 120 per minute. She was conscious with a 40 cm long sutured wound, which extended from the right ilium, passed through the medial of the right hip and ended in the right side of the anus. The patient had no sensory and motor function in her right lower extremity, and had poor peripheral circulation and extensive soft-tissue injury in her right lower abdomen and right thigh. A diagnosis of hemorrhagic shock and traumatic hemipelvectomy of the right side was made. A compressive pelvic band was used to cover the wounds. Following active resuscitation rescue, pelvic angiography was performed 6 hours after admission. Angiography showed that the right external iliac and partial internal iliac arteries were transected and occluded by thrombi, and no obvious bleeding was observed. To reduce blood loss during the amputation, the left internal iliac artery was embolized. Because of the extensive soft tissue injury and injury of the iliac artery, salvage of the injured leg is impossible. Right hemipelvectomy was performed and the wound was closed 14 hours after admission. Because of severe wound infection, debridement was required every 3 or 4 days. A total of eleven debridement and skin grafting procedures were underwent. The patient’s wound was in stable condition, and she could walk with her crutches at a 12-month follow-up after the surgery (Figure 3).

### Case 4

**Figure 4:**
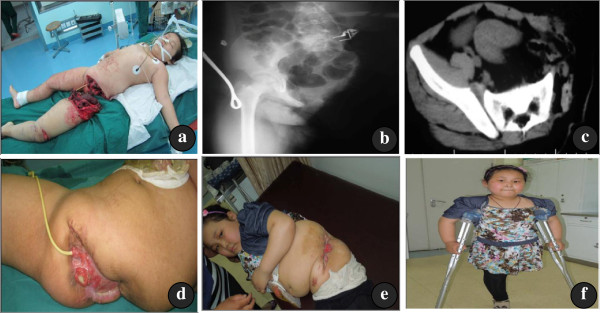
**(a) A female, 7 years old, with complete hemipelvectomy caused by a traffic accident, referred to our hospital 6 h later. (b)** The CT scan, and **(c)** X-ray film of the pelvis after the injury. Debridement was performed and the wound was left open. **(d)** By postoperative day 45, the majority of wound had healed. **(e)** &**(f)** The overview of the wound at the eighth postoperative month. The prolapse of the anal mucous membrane was noticed. The patient could ambulate with crutches.

A 7-year-old girl, injured by an oncoming vehicle while she was on a tricycle, was admitted to the local hospital with a complete traumatic amputation of her left lower limb for 5 h. She was transferred to our hospital after pressure dressing of the wound and transfusion in the local hospital. Physical examination indicated that the patient’s blood pressure was 80/50 mmHg and the pulse rate was 128 per minute. She was conscious, and her left lower extremity below the hip completely lost and the wound dressing unopened. Her blood pressure rose to 100/60 mmHg after transfusion. Debridement was performed 2 h after admission. During the surgery, we noticed that the left lower extremity beyond inguinal area was totally absent, and the area from the sacroiliac joint to the pubic bone was exposed. There were leaves and other foreign matter in the wound. The rectum, vagina and the bladder were exposed, and there was a 4 cm long laceration of the anal sphincter. The wound did not communicate with the abdominal cavity and there was no injury to the viscera. Left iliopsoas were completely avulsed from the original site and a large cavity was present in the retroperitoneal space below the diaphragm. The left common iliac artery and vein was transected and thrombosed 1 cm distal to its origin. Debridement was performed, the proximal ends of the left common iliac artery and vein were ligated and the wound was washed repeatedly. Due to the extensive skin loss, the wound was partly closed and the rest left open and covered with Vaseline Gauze after operation. Diverting colostomy was performed and thorough irrigation of the distal colon segment was conducted to prevent continued fecal contamination of the pelvic wound. The wound remained open initially, the granulation tissue gradually matured and the wound became self-contracted and closed spontaneously 6 weeks after the injury. The patient could walk with her crutches at the 12-month follow-up after the procedure (Figure 4).

## Discussion

Traumatic hemipelvectomy is a special type of pelvic fracture that is characterized by the wide separation of the innominate bone from the pubic symphysis and sacrum, the avulsion of the external iliac vessels, and the severe stretch injury or disruption of the femoral and sciatic nerves. The majority of the injuries are open fractures with extensive disruption of the soft tissues in the ipsilateral inguinal and perineal area,and greater than 50% inactivation of the injured limb when still attached to the trunk [3]. In recent years, with the improvement of medical transportation and traumatic care, the number of successfully treated patients of traumatic hemipelvectomy has gradually increased; a total of 10 cases were reported prior to 1977 [5], 19 cases till 1990 [6] and 99 cases prior to 2006.

The most common cause of the injury was a motor vehicle accident involving either pedestrians or cyclists, in which the victim was hit by an oncoming vehicle [7]. Due to the immense external forces involved, the injured limb is usually extremely rotated and dorsiflexed, resulting in complete separation of the pubic symphysis and the sacroiliac joint. Wade [8] first described this mechanism of injury in 1965, and suggested that more than 40% of traumatic hemipelvectomy cases belong to this type of injury. A second mechanism of injury involves the limbs and pelvis were entangled by heavy machinery such as the chassis of a vehicle, harvester combines or conveyor belts [3]. In addition, patients are directly injured by heavy objects, in which the upper body is thrown out of a vehicle while the legs are entangled in the car as well as the direct blow of yacht propeller, which can also result in traumatic hemipelvectomy [3,6].

The leading causes of death in patients with traumatic hemipelvectomy are blood loss, infections and multiple organ failure. The successful rescue of these patients depends on the following key steps: First, hemorrhage control and vigorous resuscitation. Direct clamping of the large bleeding vessels should be the first step in resuscitation. It has been acknowledged that circumferential compression with a sheet is cost effective method of hemostasis. Wrapping the circumference of the pelvis with sheets and knotting in front of the pelvis can form a wound compression bandage that is effective in controlling bleeding in cases with a complete separation of the injured limb from body. However, this method is sometimes less than ideal for circumstances in which the injured limb is still partially attached to the trunk. In such cases, hemipelvectomy is a life-saving intervention. It has been reported that the early angiography and subsequent embolization should be considered in cases of continued unexplained blood loss.

The hemostatic effect of artery embolization in various pelvic fractures remains controversial [9]. We reviewed 19 cases with traumatic hemipelvectomy reported from 1983 to 2005 [1,3], only four cases underwent arterial embolization before amputation and one case received artery embolization after amputation [6]. Some researchers were of the opinion that only 20% of pelvic fracture bleeding was caused by injury to the small arteries of the pelvis, other sources of bleeding included cancellous bony site and venous plexus in the pelvis [10,11]. Therefore, it suggested that arterial embolization might not be effective in stopping the majority of hemorrhage. Arterial embolization was given in 2 cases in our study. Though the wounds in the other two cases were large, there was no evidence of active bleeding, arterial embolization was not performed. and amputation surgery was carried out successfully.

The second important factor for the successful rescue of traumatic hemipelvectomy patients is early amputation. Early amputation in these patients can achieve complete hemostasis of the wound, simplify the treatment process and reduce infection and other complications. According to the principle of damage control, severe trauma and bleeding (the first strike) cause a severe inflammation and response syndrome (SIRS). Surgery and blood transfusion can act as a secondary strike which may aggravate the inflammation and result in uncontrolled systemic inflammatory response syndrome, further developing to multiple organ dysfunction syndrome (MODS), which is the main cause of delayed death in severe trauma patients. Amputation is a life-saving surgery and the surgical process should be simplified to minimize the “second hit” to the patients as long as it achieves the aim of amputation and hemostasis [9]. The surgery should be terminated immediately when the trauma triad of death viz. hypothermia (T < 35°C), coagulopathy (PT, APTT > 1.5 times of normal value) and acidosis (pH < 7.2) occur. In our experience, the primary procedure should be limited to 90 min, extensive debridement should not be attempted and the wound should be pressure dressed after surgery. It is often futile to attempt limb salvage. Pohlemann [12] attempted to salvage the limb in four cases of traumatic hemipelvectomy, 3 of them died and the remaining case had to eventually undergo amputation. Up to now, there was only one case of closed traumatic hemipelvectomy, reported by Osti [2], in which a partial success with limb salvage was achieved. However, this patient underwent below knee amputation as a result of muscle necrosis, and the remaining stump had neither sensory nor motor function. Encouragingly, the patient could wear prosthetics on the stump and walk independently without crutches.

The third most important factor for successful rescue is the treatment of associated injuries. Because the physical forces causing traumatic hemipelvectomy are tremendous, 60% of the patients sustain anorectal lesions, and 85% have genitourinary injury [3]. 48.3% and 13.8% of patiemts had other ipsilateral limb injuries and abdominal organ damage, respectively. Therefore, many researchers suggested that laparotomy should be performed as a routine step of treatment [13]. Moore [7] found missed splenic injury in a case and underwent splenectomy in the laparotomy. Colostomy should be performed in patients with anorectal injury to prevent fecal contamination of the pelvic wound. Many researchers suggested that sigmoid colostomy should also be performed in patients without anorectal injury to prevent the contamination of the pelvic wound with feces. We suggested the stoma of the colostomy should be located in the ipsilateral side of the injured leg, so the contralateral vertical rectus abdominis musculocutaneous(VARM) flap which is a life-boat flap could be used to construct the nonhealing hemipelvectomy wounds. Horst [13] summarized 59 cases of traumatic hemipelvectomy, of which 79.7% underwent sigmoid colostomy. In the four cases reported here, only one patient underwent a routine colostomy, while the remaining three cases, including one with anorectal injury, did not undergo colostomy. Wound infection developed in all four cases, and contamination and existing necrotic tissue were the main source of severe infection rather than the fecal contamination. Retrograde cystourethrogram can be used in the diagnosis of bladder and urethral injury. The treatment option is cystostomy or delayed reconstruction of the urethra, because of the higher failure rate of early reconstruction.

The fourth important factor for the rescue of these patients is repeated debridement and control of infection. Wound infection, which may result from incomplete debridement or contamination with feces and urine, is a most common cause of delayed death after traumatic hemipelvectomy [3]. The best way to prevent and treat infection is repeated debridement. Patients with serious trauma cannot tolerate long periods of extensive debridement. In addition, injured soft tissue which appears normally at early stages may become necrotic gradually. For these two reasons, repeated debridement is unavoidable. Horst’s results [13] indicated that 86% of patients underwent debridement on an average of 3.2 times, ranging from 1 to 10 times. Only in small number of cases, the wound can be closed at the primary stage followed amputation, and did not need repeated debridement [6]. All four cases reported here developed infection and presented with sustained high fever. The fever gradually decreased to normal and the wound eventually healed after repeated debridements (at least one debridement and up to eight times, the average times of debridement were five) and treatment with specific antibiotics. The timing of debridement is very important as well. The interval of debridement ranged from 3–4 day following the primary surgery. For patients whose general condition was relatively stable with normal coagulation, debridement should be more thoroughly for less bleeding. In addition, the systemic inflammatory response caused by first-strike was decreasing gradually, therefore debridement on the third or fourth postoperative day should avoid overlap of first strike with the “second hit” to prevent an excessive systemic inflammatory response. The duration of the first and second debridements should generally be limited to 90 min. Active bleeding should be stemmed before wound dressing. Hemostasis with gauze packing in this condition was unreliable, due to the extent of the wounds, the pressure dressings were unreliable and prone to loosen, resulting in incomplete hemostasis and persistent postoperative bleeding.

Particular attention should be paid to the necrosis of iliopsoas muscle during debridement. The level of iliopsoas muscle necrosis gradually rose in two patients of this group following debridement, and ultimately the iliopsoas had to be completely resected below the diaphragm. Several researchers have previously noted this phenomenon of delayed necrosis of the iliopsoas muscle [3,14]. It has been suggested that the iliopsoas undergoes strong contraction during the incident, and its blood supply was impacted significantly, which was followed by gradual necrosis. Therefore, many experts have advocated that the iliopsoas should be resected completely if there is any question about the viability of the muscle. The wound should be best covered by the myocutaneous gluteus flap after debridement. Split-thickness skin grafting can be used to cover the remaining wound if it cannot be completely covered by the gluteal flap. A free flap can also be used to cover the wound, however, this requires excellent microsurgical technique.

A patient described in another study developed Gram-negative meningitis [14]. The authors estimated that meningitis was secondary to ascending infection along the course of the avulsed lumbar and sacral nerve roots. The patient had a high fever and suffered from delirium. Gram-negative bacteria were cultured from cerebrospinal fluid. Therefore, cerebrospinal fluid culture should be considered for patients with unexplained fever and consciousness disorders after injury.

Furthermore, nutritional support, early psychiatric consultation, management of depression and phantom limb pain and timely physiotherapy contributed significantly to the functional rehabilitation of these patients. It is noteworthy that the treatment of traumatic hemipelvectomy is a very complex procedure associated with a high cost burden, which the patients and their families must be aware of.

In summary, the successful management of patients with traumatic hemipelvectomy is challenging. The principles of damage control should be adhered to in the treatment procedure. Resuscitation, hemorrhage control and amputation should be the priorities, followed by repeated debridements and wound closure. The cooperation and dedication of a multi-disciplinary team of medical staff is a prerequisite for successful treatment.

### Consent

Written informed consent was obtained from the patient for publication of this case report and any accompanying images. A copy of the written consent is available for review by the Editor-in-Chief of this journal.

## Abbreviations

RBCs: Red blood cells; SIRS: Severe inflammation and response syndrome; MODS: Multiple organ dysfunction syndrome; DIC: Disseminated intravascular coagulation.

## Competing interests

The authors report no conflicts of interest. The authors alone are responsible for the content and writing of the paper.

## Authors’ contributions

WTH: took part in the operations of the four cases and wrote the manuscript. WXR: operator of case 4 and drafted the related part. ZX: took part in the operations of cases 1-3 and took photos, drafted the manuscript. WCS: took part in the operations of cases 1-3, drafted the manuscript. ZYZ: took part in the operations of the four cases, drafted the manuscript. PAQ: operator of case 1, 2, 3 and drafted the manuscript. All authors read and approved the final manuscript.
